# Comparison of color discrimination in chronic heavy smokers and healthy subjects

**DOI:** 10.12688/f1000research.10714.3

**Published:** 2017-08-01

**Authors:** Thiago Monteiro de Paiva Fernandes, Natalia Leandro Almeida, Natanael Antonio dos Santos

**Affiliations:** 1 Department of Psychology, Federal University of Paraiba, Joao Pessoa, Brazil; 2 Perception, Neuroscience and Behavior Laboratory, Federal University of Paraíba, Joao Pessoa, Brazil

**Keywords:** cigarette smoking, visual system, color discrimination, color vision, Cambridge Colour Test, Trivector, nicotinic receptors

## Abstract

*Background: *Cigarette smoke is probably the most significant source of exposure to toxic chemicals for humans, involving health-damaging components, such as nicotine, hydrogen cyanide and formaldehyde. The aim of the present study was to assess the influence of chronic heavy smoking on color discrimination (CD). *Methods: *All subjects were free of any neuropsychiatric disorder, identifiable ocular disease and had normal acuity. No abnormalities were detected in the fundoscopic examination and in the optical coherence tomography exam. We assessed color vision for healthy heavy smokers ( *n* = 15; age range, 20-45 years), deprived smokers ( *n* = 15, age range 20-45 years) and healthy non-smokers ( *n* = 15; age range, 20-45 years), using the psychophysical forced-choice method. All groups were matched for gender and education level. In this test, the volunteers had to choose the pseudoisochromatic stimulus containing a test frequency at four directions (e.g., up, down, right and left) in the subtest of Cambridge Colour Test (CCT): Trivector. *Results: *Performance on CCT differed between groups, and the observed pattern was that smokers had lower discrimination compared to non-smokers. In addition, deprived smokers presented lower discrimination to smokers and non-smokers. Contrary to expectation, the largest differences were observed for medium and long wavelengths. *Conclusions: *These results suggests that cigarette smoking, chronic exposure to its compounds, and withdrawal from nicotine affect color discrimination. This highlights the importance of understanding the diverse effects of nicotine on attentional bias.

## Introduction

Cigarette smoking is still a major source of exposure to chemicals that are toxic for humans. The compounds in cigarettes and cigarette smoke, such as nicotine, oxygen dioxide and formaldehyde, are highly harmful to health ^1^. Data from the World Health Organization (WHO) hypothesize that by 2030, cigarettes could kill nearly 9 million people a year around the world ^2, 3^.

Cigarette nicotine deprivation in chronic users may impair cognitive and attentional abilities even after long time of cessation ^4, 5^. The neurotoxic effects of chronic use and smoking abstinence on the nervous system have not been extensively studied ^6– 8^. However, chronic cigarette smoking increases cardiovascular response ^9^, which, in turn, affects retinal responses through altered blood flow ^10^. In addition, tobacco compounds may increase free radical that would cause macular degeneration along with the action of ischemia ^11^. Whereas smoking effects on color vision are understudied, the existing data are controversial and highlights the importance of a rigorous testing procedure that measures color discrimination ^12, 13^. Thus, to identify the mechanisms underlying neurotoxic smoking effects on multisensory integration, we need to understand how smoking may alter early visual processing.

A visual percept may consist of stimuli that vary over the space (spatial contrast), time (temporal contrast) or direction of motion, and vary in luminance (achromatic) and chromaticity (saturation and hue color) ^12, 14, 15^. Thus, chromatic contrast involves chromaticity differences, which can be expressed by the distance in the CIE 1976 uniform chromaticity scale diagram and assessed by thresholds of vectors on the Cambridge Color Test (CCT), for example ^16, 17^. It has the advantage of being used to evaluate in detail whether these anomalies are due to congenital factors or acquired conditions ^16, 17^.

We base our rationale on the premise that chronic exposure to nicotine will led to receptor desensitization and not suffer influence of arousal and increase in attentional resources in smokers ^18^. The purpose of the present study was to assess the influence of chronic heavy smoking on color discrimination (CD).

## Methods

### Participants

In this study, 15 non-smokers (mean age = 32.5 years; *SD* = 9.1; 7 male), 15 cigarette smokers (mean age = 32.1 years; *SD* = 5.7; 7 male) and 15 deprived smokers (mean age = 31.9 years; *SD* = 6.3; 7 male) who were staff or students at the Federal University of Paraiba were recruited through newspaper advertisements. The participants were 25–45 years old. The participants were excluded if they had any one of the following criteria: younger than 20 and older than 45 years (since effects of aging in the human visual system could superestimate the results ^19, 20^); current history of neurological or cardiovascular disease; a history of head trauma, color blindness, current or previous drug abuse and current use of medications that may affect visual processing and cognition. Subjects were required to have good ocular health, with no abnormalities on fundoscopic examination or optical coherence tomography examination. All of the subjects were screened for color blindness using the test of Ishihara for color deficiency, and had normal or corrected-to-normal vision as determined by a visual acuity of at least 20/20.

Smokers reported a smoking history of at least 8 years, currently smoked > 20 cigarettes/day and had a score > 5 on the Fagerstrom Test for Nicotine Dependence (FTND) ^21^. Generally, smokers and deprived smokers began smoking at an average of 16.5 years of age ( *SD* = 3.25) and had been smoking for an average 15 years ( *SD* = 6.45). Smokers were allowed to smoke until the beginning of experiment. An abstinence period of 6 h was chosen based on previous studies (Bailey, Goedeker, & Tiffany, 2010; Fernandes, Almeida, & Santos, 2017; Kunchulia, Pilz, & Herzog, 2014). Non-smokers had never smoked a cigarette. No significant differences were found between depression and anxiety symptoms before and after the study, as measured by the Hamilton Scale for Depression and Hamilton Anxiety Rating Scale.

This research followed the ethical principles from the Declaration of Helsinki and was approved by the Committee of Ethics in Research of the Health Sciences Center of Federal University da Paraiba (CAAE: 60944816.3.0000.5188). Written informed consent was obtained from all participants.

### Color discrimination test

The stimuli were presented on a 19-inch LG CRT monitor with 1024 × 786 resolution and a sampling rate of 100 Hz. Stimuli were generated using a VSG 2/5 video card (Cambridge Research Systems), which was run on a microcomputer Precision T3500 with W3530 graphics card. All of the procedures were performed in a room at 26°C ± 1°C, with the walls covered in gray for better control of luminance during the experiments. All of the measurements were performed with binocular vision. Monitor luminance and chromatic calibrations were performed with a ColorCAL MKII photometer (Cambridge Research Systems).

The color vision test was performed using CCT, version 2.0, with Trivector subtest (Cambridge Research Systems; http://www.crsltd.com/tools-for-vision-science/measuring-visual-functions/cambridge-colour-test/). The CTT was performed in a darkened room with illumination that was provided only by the monitor that was used to present the visual stimuli. Trivector provides a clinical assessment of color vision deficiencies as a rapid means screening of the existence of congenital or acquired deficits ^16^. CCT uses pseudoisochromatic stimuli (Landolt C) defined by the test colors that are to be discriminated, on an achromatic background. The figure and the background are composed of grouped circles randomly varying in diameter and having no spatial structure (variation of 5.7° arcmin of external diameter and 2.8° arcmin of internal diameter). The luminance variation in each response avoids learning effects or use of tricks to respond correctly.

The four-alternative forced-choice ^16, 22^ (4-AFC) method was used, and the subjects’ task was to identify, using a remote control response box, whether the Landolt ‘C’ stimulus was presented at the left, right, up or down side of the monitor screen. The participants were also instructed to respond whether they could not identify the stimulus gap ^16^. After each correct answer, the chromaticity of the target proceeded closer to that of background, while each wrong answer or omission was followed by the presentation of the target at a greater chromatic distance from the background. After each correct answer, the chromaticity of the target proceeded closer to the background. Each incorrect answer or omission was followed by presentation of the target at a greater chromatic distance from the background. The experiment ended after 11 reversals for each axis and the threshold was estimated from the six final reversals ^23^.

The trivector testing protocol estimates sensitivity for the short, medium and long wavelengths through the protanopic, deuteranopic, and tritanopic confusion axes, respectively ^23, 24^. Trivector protocol uses vectors as central measurement. The advantage of this brief test is that it can be performed in about 5 minutes and provides a reliable result ^16^. The three confusion axes converge at a co-punctual point, and the u’v’ coordinates (CIE 1976) used were: protan (0.6579, 0.5013), deutan (-1.2174, 0.7826) and tritan (0.2573, 0.0000) (for more details, see 17).

In general, we used a default setting where the Landolt ‘C’ had an opening at 1° of visual angle, minimum luminance of 8 cd/m², maximum luminance of 18 cd/m², 6 s of response time for each trial and distance of 269 cm between participant and monitor screen.

### Data analysis

The distributions for each group were compared with Shapiro-Wilk. Both groups showed non-normal distribution; therefore, nonparametric statistical methods were used to analyze the data. For group comparisons, the non-parametric univariate analysis was used, with pairwise comparisons by Mann-Whitney *U* test. Spearman’s rank correlation coefficients (rho) were conducted to assess the relationship between outcomes of color discrimination data and biosociodemographic variables, such as age, gender and level of education. All the calculations were made using SPSS ^®^, version 21.0.

The effect size ( *r*) estimation was used from the conversion of z-scores ^25, 26^. Values above .50 are considered as large effect size.

Results are presented as medians. Center lines show the medians; box limits indicate the 25th and 75th percentiles as determined by SPSS software; whiskers extend 1.5 times the interquartile range from the 25th and 75th percentiles (ends of the whiskers are the maximum and minimum values). When presented, errors bars represent standard deviations (SD) of the median based on 1000 bootstrap resamplings. Bonferroni correction was the method of adjusting the *P*-value that we used. *P* < 0.016 was accepted as statistically significant for multiple comparisons and *P* < 0.025 for pairwise comparisons.

## Results

**Figure 1.**
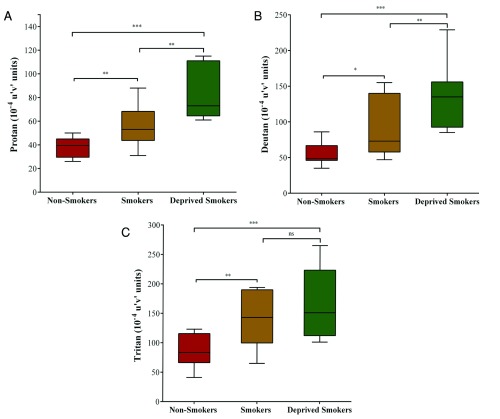
Trivector test: box-and-whiskers plots for protan ( **A**), deutan ( **B**) and tritan ( **C**) confusion lines. Data are presented in 10 ^-4^ u’v’ units. Each box-and-whiskers plot is based on results for 45 participants. * *P* < 0.05; ** *P* < 0.01; *** *P* < 0.001.

Color discrimination thresholds were obtained in u'v' units of the CIE 1976 color diagram, for protan, deutan, and tritan axes, respectively. Nonparametric analysis were carried out showing that there were significant differences in discrimination thresholds between groups along the protan (χ²(2) = 26.53, *P* < 0.001), deutan (χ²(2) = 22.40, *P* < 0.001) and tritan (χ²(2) = 14.93, *P* < 0.001) confusion axes. Thresholds for the smokers and deprived smokers were higher than the normative values observed in other studies. Therefore, there was a reduction in color discrimination in both groups. The results of the trivector measurements are shown in Figure 1.

Along protan vectors ( Figure 1A), pairwise comparisons showed that discrimination thresholds were higher in the group of smokers compared to non-smokers ( *U* = 132, *P* = 0.002, *r* = -.61). In addition, deprived smokers had the highest thresholds compared to the group of non-smokers ( *U* = 105, *P* < 0.001, *r* = -.85) and smokers ( *U* = 136, *P* = 0.002, *r* = -.58).

Along deutan vectors ( Figure 1B), when compared with the control group, smokers ( *U* = 136, *P* = 0.001, *r* = -.58) and deprived smokers ( *U* = 108, *P* < 0.001, *r* = -.83) presented higher discrimination thresholds, with high effect size. There was statistically significant differences between smokers and deprived smokers ( *U* = 154, *P* = 0.024, *r* = -.43).

Along tritan vectors ( Figure 1C), when compared with the control group, smokers ( *U* = 140, *P* = 0.003, *r* = -.55) and deprived smokers ( *U* = 126, *P* < 0.001, *r* = -.67) presented higher discrimination thresholds. There was no statistically significant differences among smokers vs. deprived smokers ( *P* = 0.250).

### Correlations

There is no relationship between color discrimination and gender (chi-square = 72, df = 39, *P* > 0.05). A spearman correlation showed no correlation between FTND and trivector data *(P* > 0.050), color discrimination and education years [rho = .078, *P* = 0.515], and color discrimination and age [rho = .096, *P* = 0.347].

Patient demographics and Trivector results Click here for additional data file. Copyright: © 2017 Fernandes TMdP et al. 2017 Data associated with the article are available under the terms of the Creative Commons Zero "No rights reserved" data waiver (CC0 1.0 Public domain dedication).

## Discussion

The data indicated that smokers groups, as a whole, had higher discrimination thresholds when compared to non-smokers ( *P* < 0.05), indicating the existence of a diffuse impairment in visual processing. Results showed good agreement between the normative data of control groups, being the protan and deutan thresholds lower than tritan thresholds, a pattern repeatedly observed in adults tested with the CCT ^17^. Moreover, the higher thresholds observed in the group of smokers and deprived smokers are in agreement with the differences observed in other studies using CCT. The effect sizes reached medium to high values.

Small differences in blue-yellow color processing suggest that sensor neurons responsive to the short wavelength may differently operate from those responding to medium and long wavelengths ^27^. Indeed, the koniocelular pathway may not suffer from the influences of tobacco components.

Along the trivector protocol, smokers had more errors in protan and deutan confusion axes ( Figure 1). An effect size analysis confirmed that smokers had the largest discrimination errors for protan ( *r* = -85) and deutan ( *r* = -82) confusion axes when comparing against non-smokers. As stated, this result does not support the idea of channel selectivity. However, we base our rational on the existence of diffuse processing impairment, which may include magno- and parvocellular pathways ^28^.

Nicotine enhances neurotransmission release through modulation of nicotinic acetylcholine receptors (nAChRs) located in the cortex ^18, 29^. There are also nAChRs and dopamine receptors on the retina; therefore, the chronic use of cigarette would enhance attentional resources ^30– 32^. However, there were no improvements in color discrimination. One may argue this could be due to desensitization effect, one of many brain changes caused by addiction ^33^. Chronic nicotine exposure leads to nAChRs desensitization through brain upregulation ^34, 35^. The more exposure, the greater the need for it activate the receptors ^36, 37^. Whereas nicotine enhancing effects remain unchanged after chronic exposure, this may explain the lower discrimination, but the small similarity, between smokers and non-smokers in some of our data ( Figure 1).

Then, why did the deprived smokers group have less discrimination? The withdrawal effect, which affect neurotransmission release ^38, 39^, reflecting both visual processing ^40– 42^ and brain reward function ^43^, may explain this. Visual attention plays a role for detection of environmental stimuli ^44^.

As stated, impairments observed at color discrimination can occur due to cones saturation, amplification of the noise that reach visual cortex or by the action of nicotine in parvocelular pathway ^45^. In agreement with studies, color vision impairments may be related to ventral stream, which processes color ^46^. However, our tests used pseudoisochromatic stimuli. Thus, color discrimination may have occurred through dorsal and ventral stream ^28^. Too soon to conclude anything, but there may be nAChRs in both dorsal and ventral stream ^47^ and both streams may suffer from the action of neurotrasmission hypofunction, affecting directly visual processing ^39– 41, 43^.

Knowing the existence of the expression of nAChRs in bipolar, amacrine and ganglionar cells ^29, 47, 48^, we suggest that smoking affects visual processing, regardless of deprivation. Although the differences between smokers and non-smokers were small, we could not ignore the existence of many harmful compounds to vision in cigarettes. As noted in others studies, exposure to cigarette smoking ^49– 55^ and solvents ^56, 57^ affects vision. Thus, smoking can be harmful even for passive smokers.

Our limitations need to be considered. We evaluated cigarette smoking as a whole, not the nicotine-only effects ^51, 52^. Which brings us to the idea of further studies, using nicotine gum and the same paradigm used here. Clearly, further work is needed, but this study highlights the relationship between smoking and color discrimination, involving short, medium and long wavelengths ^27^. We conclude that cigarette compounds affect vision ^54, 55^ more than nicotine separately ^58– 61^.

## Data availability

The data referenced by this article are under copyright with the following copyright statement: Copyright: © 2017 Fernandes TMdP et al.

Data associated with the article are available under the terms of the Creative Commons Zero "No rights reserved" data waiver (CC0 1.0 Public domain dedication). 

**Dataset 1: Patient demographics and Trivector results.** Raw data of the subjects biosociodemographic and trivector (protan, deutan and tritan) results. doi, 10.5256/f1000research.10714.d150059 ^62^
