# Supplementary material for: Comparison of color discrimination in chronic heavy smokers and healthy subjects
Source: F1000Res. 2017 Aug 1;6:85. Originally published 2017 Jan 27. [Version 3] doi: 10.12688/f1000research.10714.3 (PMC5580434; doi:10.12688/f1000research.10714.3)
Supplement: Peer review history

# Peer review: Referee response for version 3

**DOI:** 10.5256/f1000research.13276.r24707

**Published:** 2017-08-07

**Authors:** Goro Maehara

**Affiliations:** 1 Department of Human Sciences, Kanagawa University, Yokohama, Japan

The authors have addressed all my concerns. Thanks.

I have read this submission. I believe that I have an appropriate level of expertise to confirm that it is of an acceptable scientific standard.

# Peer review: Referee response for version 2

**DOI:** 10.5256/f1000research.12149.r21404

**Published:** 2017-04-27

**Authors:** Marine Raquel Diniz da Rosa

**Affiliations:** 1 Neuroscience and Behavior Graduate Program , Federal University of Paraíba , João Pessoa, Brazil

From my point of view, the authors have made relevant changes to the paper and have met all the requests previously suggested. In a way that was reviewed by the authors, it was clarified the cut-off points used for the color discrimination, contributing to a better understanding of the results. In addition, the discussion has improved. Thus, I believe that now the paper should be accepted for publication.

I have read this submission. I believe that I have an appropriate level of expertise to confirm that it is of an acceptable scientific standard.

# Peer review: Referee response for version 2

**DOI:** 10.5256/f1000research.12149.r21405

**Published:** 2017-04-03

**Authors:** Goro Maehara

**Affiliations:** 1 Department of Human Sciences, Kanagawa University, Yokohama, Japan

I found the manuscript was improved but still have some concerns.

 1. Outcome Cutoffs

 There are some difficulties to understand the Outcome Cutoffs section. The authors should state clearly how they determined and calculated the cutoff points. I also suggest explaining what the values in parentheses represent and what IQR means (interquartile range). Although the authors mentioned ‘(mean + median + IQR + upper and lower limits)’, the plus signs in this phrase seem a bit odd.

 2. The Results Section

 Please clearly compare the thresholds with the cutoffs values. Not only the median thresholds for smokers, but those for normal observers also exceeds the cutoff values in the deutan and tritan tests, don’t they? I think that the cutoff values might be too strict (low).

I have read this submission. I believe that I have an appropriate level of expertise to confirm that it is of an acceptable scientific standard, however I have significant reservations, as outlined above.

# Response

**Published:** 2017-04-03

**Authors:** Thiago P Fernandes

**Affiliations:** Federal University of Paraiba, Brazil

Adding the cutoff values ​​was just one way to meet the review specifications. If you take a brief look at all papers using Trivector as test, there are no specifications for cut-off values. However, the paper of Paramei *et al.* (2012) compared the color discrimination across four life decades, and used data obtained in Brazil. If it is possible, at least briefly, to read this and other papers using Trivector, you will notice a little of what I am talking about.

 In addition, when reading other articles about Trivector, you will observe a simple pattern: **generally, both the control and the observed (experimental) conditions may have values below 100** for the protan or deutan (eg) confusion axes. The values ​​obtained by Mollon & Reffin **unfortunately do not match experimental practice**. The reasons are more varied: type of computer, type of processor used at the time, condition of luminance or characteristic of the study. The following Mollon & Reffin studies used the commercial version called Cambridge Color Test, where we tried to standardize and avoid differences between studies. This standardization could be observed when Paramei *et al.* (2012) **observed that there are not many differences in results for controls both in Brazil and around the world.**

 No matter how small the visual processing loss is, the differences that will be classified as impairments will be the statistically significant differences. If it was not clear, I will summarize. Conjecturing: If in one study, for the protan axis, the control group had 50 10 ^-4^ u' v' units and the experimental one had 80 10 ^-4^ u' v' units, having a value of p <.001, this is considered color impairment. Thus, the experimental group would present damage in color processing. **This is what indicates most, if not 90% of all articles that used the Trivector as a measure.**

 I hope it has helped in understanding how the Trivector test works. Even so, i  will try to answer your questions below:

 1. Outcome Cutoffs

- ***There are some difficulties to understand the Outcome Cutoffs section. The authors should state clearly how they determined and calculated the cutoff points.***

This was explained. The mean or median (+ SD or + IQR) for the control group of all the studies that used the Trivector were summed and divided by the number of studies (a simple grand mean / median).

- ***I also suggest explaining what the values in parentheses represent and what IQR means (interquartile range). ***

*"Based on previous studies, cutoff points (median + IQR/2) for the trivector test were established to designate color vision impairment.*" As stated, median + IQR/2 was established by Paramei *et al.* (2012). This was also explained. In my opinion, there is no need to explain why using IQR/2 or IQR, since they are simply representing the standard deviation in a non-parametric data

  

- ***Although the authors mentioned ‘(mean + median + IQR + upper and lower limits)’, the plus signs in this phrase seem a bit odd.*** 

I understand your point of view. However, the reason for inserting this comment into the text was to explain to the reader that there are normative values for the Trivector. These values would be means, medians, tolerance values, etc. They can be observed in the reference article quoted in the cutoffs outcome section.

 2. The Results Section

- ***Please clearly compare the thresholds with the cutoffs values. Not only the median thresholds for smokers, but those for normal observers also exceeds the cutoff values in the deutan and tritan tests, don’t they? I think that the cutoff values might be too strict (low). ***

As explained, the use of cutoff values was only one way to meet the requests of the reviewers. However, in no study using trivector cutoff values are imposed. This is due to the fact that to consider impairment in color vision it is only necessary to have statistically significant differences and to have medium or high effect size.

 Nevertheless, the medians of our results for the control group are "beating" with the cutoff values + IQR/2.

 So, the purpose of this comment was to try to show how the trivector works. Just read one or two papers that made use of the test and you will note that the differences need not exceed the set values of 100, 100 and 150 10 ^-4^ u' v 'units to prove impairments at color vision. Even differences observed in values smaller than 100 10 ^-4^ u'v 'units are significant differences, since this is a robust and reliable test.

 According to Trivector's studies, there is no large or small differences, there is statistically significant differences, with large or small effect sizes. And this was what we observed in our study.

 Thank you for the review.

 Kind Regards,

# Response

**Published:** 2017-06-06

**Authors:** Goro Maehara

**Affiliations:** Kanagawa University, Japan

Dear Thiago,

The authors have solved the major concern in my 1st response through the revision process. Mollon & Regan (2000) set the cutoff values relatively high probably because the test was originally designed for detecting color blindness.

Here we need to tackle the issues about the present cutoff values.

I agree with another reviewer in that the standard of normality is useful for making a comparison between the present and previous studies. Any analysis in a paper must be logical even if authors think a specific analysis is not very important. In the present study, not only the thresholds for the smoker, but also those for normal controls exceeded the cutoff values for the tritan test (and deutan test, too?). The authors need to somehow solve this logical problem.

Thank you for the explanations on the cutoff values. That is, the cutoff values were defined as the average of normal observers’ (median threshold + IQR/2) reported by the previous studies (or only by Paramei?). It will be helpful for readers to rewrite the Outcome Cutoff section as the authors commented above.

The cutoff values of (median threshold + IQR/2) mean that 25 % of the population is classified as abnormal. It seems reasonable for me to increase the cutoff values because 25 % of the population is too much. This might be a possible solution.

Another possible solution could be discussing why the thresholds for normal observers exceeded the cutoff values in the Discussion section. The authors could also emphasize the importance of statistical differences there.

If the authors have any idea for workable solutions, please feel free to let me know.

Minor concerns,

1. My previous comment on IQR was misleading. I would like to suggest inserting ‘( interquartile range)’ just after IQR when the authors first use the abbreviation.
2. If the authors used data reported by researchers other than Paramei (2012) for the cutoff value calculation, I suggest including the citations of the studies.

 

Best wishes,

Goro

# Peer review: Referee response for version 1

**DOI:** 10.5256/f1000research.11553.r20562

**Published:** 2017-03-17

**Authors:** Marine Raquel Diniz da Rosa

**Affiliations:** 1 Neuroscience and Behavior Graduate Program , Federal University of Paraíba , João Pessoa, Brazil

The article investigates and compares color discrimination in chronic smokers and healthy individuals. The authors found a lower significant color discrimination in chronic smokers.

 However, I believe that in order to clarify the cut-off point of color discrimination, authors should write in Methods the standard of normality used to rate low or high color discrimination. In addition, in the description of the results, for better understanding, the authors should explain better the results (which are below the expected) and then the significance of them.

 The results suggest a possible, even small, important change to the color discrimination of smokers  which deserves attention and should be better studied. Therefore, I believe that the paper should be accepted for publication with the aforementioned suggestions.

I have read this submission. I believe that I have an appropriate level of expertise to confirm that it is of an acceptable scientific standard, however I have significant reservations, as outlined above.

## References

[1] MollonJDReganBC: Cambridge Colour Test Handbook. *Cambridge Research Systems Ltd.*.2000;

[2] GoulartPRK: A computer-controlled color vision test for children and non-verbal subjects based on the Mollo-Reffin color discrimination test. *Doctoral Dissertation,Universidade Federal do Pará*.2008;75

[3] OliveiraA.R: Chromatic and achromatic psychophysical evaluations of men and women exposed to organic solvents. *Master Dissertation Universidade Federal da Paraíba*.2015;75

[4] GoulartPRBandeiraMLTsubotaDOiwaNNCostaMFVenturaDF: A computer-controlled color vision test for children based on the Cambridge Colour Test. *Vis Neurosci*.25(3) : 10.1017/S0952523808080589 445-50 10.1017/S0952523808080589 18598416

# Response

**Published:** 2017-03-24

**Authors:** Thiago P Fernandes

**Affiliations:** Federal University of Paraiba, Brazil

Dear Marine,

 First of all, many thanks for the reading and suggestions for our manuscript.

 We will try to answer your questions below:

  

- *However, I believe that in order to clarify the cut-off point of color discrimination, authors should write in Methods the standard of normality used to rate low or high color discrimination. *

We appreciate the suggestion. These changes were made. We stand by calculating the average of the control group of all studies using Trivector in Brazil and using the normative data for age groups used by Paramei et al. ^1^

- *In addition, in the description of the results, for better understanding, the authors should explain better the results (which are below the expected) and then the significance of them. *

We appreciate the suggestion. Although the description of Trivector's results is quite directive (for details, see ^2-5^), we agreed that the way the results were presented was below expectations.These changes were made.

  

- *The results suggest a possible, even small, important change to the color discrimination of smokers  which deserves attention and should be better studied. *

In agreement. Based on this suggestion, we added a few paragraphs on the importance of the present study, see Introduction and Discussion.

**References:**

  

1. Paramei GV. Color discrimination across four life decades assessed by the Cambridge Colour Test. *J Opt Soc Am A Opt Image Sci Vis*. 2012;29(2):A290-297.
2. Lacerda EM da CB, Ventura DF, Silveira LC de L: Visual assessment by psychophysical methods of people subjected to occupational exposure to organic solvents. *Psicol USP.* 2011;22(1):117–145. 10.1590/S0103-65642011005000011
3. Oliveira AR: Avaliações psicofísicas cromática e acromática de homens e mulheres expostos a solventes orgânicos.2015. Reference Source
4. Goulart PRK, Bandeira ML, Tsubota D, Oiwa NN, Costa MF, Ventura DF. A computer-controlled color vision test for children based on the Cambridge Colour Test. *Vis Neurosci*. 2008;25(3):445-450. doi:10.1017/S0952523808080589.
5. Costa MF, Oliveira AGF, Feitosa-Santana C, Zatz M, Ventura DF. Red-Green Color Vision Impairment in Duchenne Muscular Dystrophy. *Am J Hum Genet*. 2007;80(6):1064-1075

# Peer review: Referee response for version 1

**DOI:** 10.5256/f1000research.11553.r20559

**Published:** 2017-03-10

**Authors:** Goro Maehara

**Affiliations:** 1 Department of Human Sciences, Kanagawa University, Yokohama, Japan

The authors measured color discrimination thresholds in chronic smokers and non-smokers using the Cambridge Color Test. The color discrimination thresholds were significantly higher for chronic smokers than non-smokers. Although their methods were scientifically sound, the thresholds for chronic smokers were not high enough to conclude that smoking impairs their color discrimination abilities.

 Major concern

 1. The thresholds for chronic smokers

 According to Mollon & Regan (2000) ^1^, “normal limits for performance for first examination on the basic “Trivector” test are 100 (protan), 100 (deutan) and 150 (tritan).” The threshold medians for chronic smokers were lower than those values, except for the deutan threshold median for deprived smokers.

 Although there were statistically significant difference between chronic smokers and non-smokers, the thresholds for non-smokers were very low (about 40, 50, and 80 in 10 ^−4^ u’ v’ units for protan, deutan, and tritan, respectively). In addition, the differences in thresholds (about 60) make little change in color appearance.

 Taken together, it seems difficult to conclude that smoking impairs color discrimination abilities.

 Minor concern

 1. Equation 1

 I am not sure why Weber contrast (equation 1) needs to be explained in the Methods section. The authors should state clearly how they used the equation in their experiment.

 2. Results

 The authors just listed the statistical results in the Results section. I suggest describing the results in the more detailed way (ex. thresholds were higher for smokers than non-smokers, *U* = 132, *P* = 0.002, *r* = -.61).

 3. 3rd paragraph in the Discussion section

 The authors stated that “smokers were more sensitive to protanopic and deutanopic confusion axes.” This sentence is confusing. Which does this sentence mean, “more sensitive than non-smokers” or “more sensitive than to tritanopic axes”?

I have read this submission. I believe that I have an appropriate level of expertise to state that I do not consider it to be of an acceptable scientific standard, for reasons outlined above.

## References

[1] MollonJDReganBC: Cambridge Colour Test Handbook (version 1.1). *Cambridge Research Systems Ltd.*.2000; Reference source

# Response

**Published:** 2017-03-10

**Authors:** Thiago P Fernandes

**Affiliations:** Federal University of Paraiba, Brazil

Dear Goro Maehara,

 We respectfully thank you for the reading and responding to our manuscript.

 If I may contest your decision of this manuscript, we respectfully do not agree that it can not be accepted as an acceptable scientific standard. We believe that the data contribute in terms of scientific validity, and because we know that there are few studies on color vision in chronic smoking (and abstinence).

 We will try to answer your questions below:

  

- *"Although their methods were scientifically sound, the thresholds for chronic smokers were not high enough to conclude that smoking impairs their color discrimination abilities."*

Although we did not observe large differences between the thresholds of the control group and the group of smokers, we agree with Lakens (2013) and Field (2013) noting that the statistically significant differences are consonant with the related effect sizes which fluctuated between mid-to-high values ( *r* values between .50 and .61; chronic smokers x controls). Moreover, when comparing the group of smokers with deprived smokers, we observed that these differences were not so large ( *r* values reaching .50). We do not know if the smoking habit, cigarette compounds or smoking *per se*, are responsible for the decrease in color discrimination.

 But there was a loss of color discrimination, suggesting the idea that visual color processing may be diffusely impaired in smokers (Besson et al., 2007; Vallejo, Buisson, Bertrand, & Green, 2005; Zhang, Dong, Doyon, & Dani, 2012). We base this hypothesis on the fact that the many cigarette compounds, including organic solvents in the cigarette smoke, impairs color processing *per se*.

**Major Concern**

- ' *The threshold medians for chronic smokers were lower than those values, except for the deutan threshold median for deprived smokers. Although there were statistically significant difference between chronic smokers and non-smokers, the thresholds for non-smokers were very low (about 40, 50, and 80 in 10−4 u’ v’ units for protan, deutan, and tritan, respectively). In addition, the differences in thresholds (about 60) make little change in color appearance. Taken together, it seems difficult to conclude that smoking impairs color discrimination abilities.'*

Many thanks for the review.

 Based on our expertise in the use of Cambridge Colour Test: **the minor the threshold, better discrimination**. If a group (in this case, smokers group) has a higher threshold, this means that they needed more chromatic contrast to detect the stimuli. **Thus, higher thresholds means lower discriminiation along confusion axes** (Hasrod & Rubin, 2015).

 After the publication of Mollon and Reffin's about the CCT (2000), several studies using Trivector have been published. Including preliminary norms for the use of CCT (Ventura et al., 2003), which was considered by the creators of the CCT on the software website (http://www.crsltd.com/tools-for-vision-science/measuring-visual-functions/cambridge-colour-test/)

 We re-checked our Trivector data and compared to several studies and we observed that we're with similar values for control groups. If another group (such smokers or deprived smokers) had higher thresholds, it means that they differ from the standard values and are likely to have color vision impairments.

 As shown in the other studies, values for control subjects have fluctuated precisely in the values that we obtained in our data (Costa et al., 2007; Goulart et al., 2008; Paramei, 2012, 2014; Ventura et al., 2002).

 Thus, the raising of the threshold of smokers is possibly connected with smoking conditions, since we've matched all possibly intervenient variables.

 Taken together, we can not ignore that, although not as large, the differences were significant in this sample. Based on previous studies, even though there are small differences, they need to be punctuated, since we agree that this is an important area that requires further research.

**Minor concern**

- *1. Equation 1; I am not sure why Weber contrast (equation 1) needs to be explained in the Methods section. The authors should state clearly how they used the equation in their experiment.*

Ops! Many thanks! Since CCT already uses this default setting, we strongly agreed with your review. These changes will be in the second version of the manuscript (we will remove it).

- *2. Results; The authors just listed the statistical results in the Results section. I suggest describing the results in the more detailed way (ex. thresholds were higher for smokers than non-smokers, U = 132, P = 0.002, r = -.61).*

Many thanks again. We believe that the way you suggested will facilitate the reader's understanding and will enhance the scientific level of our writing. We fixed it. They will be more descriptive in the next version

- *3. 3rd paragraph in the Discussion section; The authors stated that “smokers were more sensitive to protanopic and deutanopic confusion axes.” This sentence is confusing. Which does this sentence mean, “more sensitive than non-smokers” or “more sensitive than to tritanopic axes”?*

We appreciate the suggestion and agree that the use of two forms of explanation may actually confuse the reader. We will correct this.

 However, when we mention that the smoking group was more sensitive to the protanopic or deutanopic axes, we simply mean that they made more errors than the control group, for example. That is, they needed more chromatic contrast (they were more sensitive) than the comparison group. The confusion axes refer to the red (protanopic), green (deutanopic) and blue (tritanopic) axes. Thus, if any group was more sensitive to the red confusion axis, forr example, it means that they possibly had impairments in the processing of this wavelength.

 In this way, based on the apointments above, the ***'not approved'*** status is honestly inconsistent with the content of the work. We ask you to reconsider your decision and we are grateful for the comment, reading, and review of the manuscript.

**References**

 1. Lakens D. Calculating and reporting effect sizes to facilitate cumulative science: a practical primer for t-tests and ANOVAs. Front. Psychol. [Internet]. 2013 [cited 2017 Mar 10];4. Available from: http://journal.frontiersin.org/article/10.3389/fpsyg.2013.00863/abstract

 2. Field A. Discovering Statistics Using IBM SPSS Statistics. SAGE; 2013.

 3. Besson M, Granon S, Mameli-Engvall M, Cloëz-Tayarani I, Maubourguet N, Cormier A, et al. Long-term effects of chronic nicotine exposure on brain nicotinic receptors. Proc. Natl. Acad. Sci. 2007;104:8155–60.

 4. Vallejo YF, Buisson B, Bertrand D, Green WN. Chronic Nicotine Exposure Upregulates Nicotinic Receptors by a Novel Mechanism. J. Neurosci. Off. J. Soc. Neurosci. 2005;25:5563–72.

 5. Zhang L, Dong Y, Doyon WM, Dani JA. Withdrawal from Chronic Nicotine Exposure Alters Dopamine Signaling Dynamics in the Nucleus Accumbens. Biol. Psychiatry. 2012;71:184–91.

 6. Mollon JD, Regan BC. Cambridge Colour Test Handbook [Internet]. 2000. Available from: https://sites.oxy.edu/clint/physio/article/CAMBRIDGECOLOURTESTHandbook.pdf

 7. Ventura DF, Silveira LC de L, Rodrigues A, Costa MF. Preliminary Norms for the Cambridge Colour Test. 2003;331–9.

 8. Costa MF, Oliveira AGF, Feitosa-Santana C, Zatz M, Ventura DF. Red-Green Color Vision Impairment in Duchenne Muscular Dystrophy. Am. J. Hum. Genet. 2007;80:1064–75.

 9. Feitosa-Santana C, Barboni MTS, Oiwa NN, Paramei GV, Simões ALAC, Da Costa MF, et al. Irreversible color vision losses in patients with chronic mercury vapor intoxication. Vis. Neurosci. 2008;25:487–91.

 10. Goulart PRK, Bandeira ML, Tsubota D, Oiwa NN, Costa MF, Ventura DF. A computer-controlled color vision test for children based on the Cambridge Colour Test. Vis. Neurosci. 2008;25:445–50.

 11. Paramei GV. Color discrimination across four life decades assessed by the Cambridge Colour Test. J. Opt. Soc. Am. A Opt. Image Sci. Vis. 2012;29:A290-297.

 12. Paramei GV, Oakley B. Variation of color discrimination across the life span. JOSA A. 2014;31:A375–84.

 13. Pelli DG, Bex P. Measuring contrast sensitivity. Vision Res. 2013;90:10–4.

 14. Hogg RE, Chakravarthy U. Visual function and dysfunction in early and late age-related maculopathy. Prog. Retin. Eye Res. 2006;25:249–76.

 15. DeValois KK, Webster MA. Color vision. Scholarpedia. 2011;6:3073.

# Response

**Published:** 2017-03-19

**Authors:** Goro Maehara

**Affiliations:** Kanagawa University, Japan

Dear Thiago,

 I am happy to review the revised manuscript.

 Please make it clear that the thresholds of normal observers were comparable with those reported by previous studies using the Cambridge color test.

 According to Thornton, Edwards, Mitchell, Harrison, Buchan & Kelly (2005),  there is a strong association between current smoking and age-related macular degeneration. This line of studies could strengthen your paper.

 Regards,

 Goro

# Response

**Published:** 2017-03-24

**Authors:** Thiago P Fernandes

**Affiliations:** Federal University of Paraiba, Brazil

Dear Goro,

 Many thanks for the quick answer.

 Based on your suggestions, substantial changes were made.

 We inserted a subsection in the methods where we explained the cutoff points (where the results would be normal and where the discrimination losses would be).

 In addition, we better describe the results section, making it clear to the reader that the higher the threshold, the lower the color discrimination. Also, based on your last suggestion, we added a few paragraphs about the relationship between the harmful cigarette compounds and the damage they cause to the retina, and consequently, visual processing.

 I hope we have answered the suggestions. Again, we ask you to reconsider your decision about the status of our work.

 Best regards,
